# A selective p38α/β MAPK inhibitor alleviates neuropathology and cognitive impairment, and modulates microglia function in 5XFAD mouse

**DOI:** 10.1186/s13195-020-00617-2

**Published:** 2020-04-21

**Authors:** Min Sung Gee, Seung Hwan Son, Seung Ho Jeon, Jimin Do, Namkwon Kim, Yeon-Joo Ju, Soo Jin Lee, Eun Kyoung Chung, Kyung-Soo Inn, Nam-Jung Kim, Jong Kil Lee

**Affiliations:** 1 Department of Fundamental Pharmaceutical Science, Graduate School, Kyung Hee University, 26 Kyungheedae-ro, Dongdaemun-gu, Seoul, 02447 Republic of Korea; 2 Department of Biomedical Science and Technology, Graduate School, Kyung Hee University, 26 Kyungheedae-ro, Dongdaemun-gu, Seoul, 02447 Republic of Korea; 3 Department of Life and Nanopharmaceutical Sciences, Graduate School, Kyung Hee University, 26, Kyungheedae-ro, Dongdaemun-gu, Seoul, 02447 Republic of Korea; 4 Department of Pharmacy, College of Pharmacy, Kyung Hee University, 26, Kyungheedae-ro, Dongdaemun-gu, Seoul, 02447 Republic of Korea

**Keywords:** Alzheimer’s disease, Amyloid-β, P38 mitogen-activated protein kinase, Kinase inhibitor, Microglia

## Abstract

**Background:**

Chronic neuroinflammation, aggressive amyloid beta (Aβ) deposition, neuronal cell loss, and cognitive impairment are pathological presentations of Alzheimer’s disease (AD). Therefore, resolution of neuroinflammation and inhibition of Aβ-driven pathology have been suggested to be important strategies for AD therapy. Previous efforts to prevent AD progression have identified p38 mitogen-activated protein kinases (MAPKs) as a promising target for AD therapy. Recent studies showed pharmacological inhibition of p38α MAPK improved memory impairment in AD mouse models.

**Methods:**

In this study, we used an AD mouse model, 5XFAD, to explore the therapeutic potential of NJK14047 which is a novel, selective p38α/β MAPK inhibitor. The mice were injected with 2.5 mg/kg NJK14047 or vehicle every other day for 3 months. Morris water maze task and histological imaging analysis were performed. Protein and mRNA expression levels were measured using immunoblotting and qRT-PCR, respectively. In vitro studies were conducted to measure the cytotoxicity of microglia- and astrocyte-conditioned medium on primary neurons using the MTT assay and TUNEL assay.

**Results:**

NJK14047 treatment downregulated phospho-p38 MAPK levels, decreased the amount of Aβ deposits, and reduced spatial learning memory loss in 9-month-old 5XFAD mice. While the pro-inflammatory conditions were decreased, the expression of alternatively activated microglial markers and microglial phagocytic receptors was increased. Furthermore, NJK14047 treatment reduced the number of degenerating neurons labeled with Fluoro-Jade B in the brains of 5XFAD mice. The neuroprotective effect of NJK14047 was further confirmed by in vitro studies.

**Conclusion:**

Taken together, a selective p38α/β MAPK inhibitor NJK14047 successfully showed therapeutic effects for AD in 5XFAD mice. Based on our data, p38 MAPK inhibition is a potential strategy for AD therapy, suggesting NJK14047 as one of the promising candidates for AD therapeutics targeting p38 MAPKs.

## Background

Alzheimer’s disease (AD) is a common progressive neurodegenerative disorder characterized by severe neuronal loss leading to cognitive dysfunction. At present, no effective drug or treatment has been available for the prevention or cure of this disease [1–3]. The major neuropathological feature of AD is the aggregation and deposition of amyloid β (Aβ) peptides which are considered one of the major risk factors and causes of AD [3, 4]. Aggregated Aβ peptides play critical roles in neuronal degeneration, neuroinflammation, and oxidative stress [5, 6]. Thus, therapeutic approaches for the effective removal of Aβ deposits may provide neuroprotective benefits in AD.

According to recently published studies, AD may be exacerbated by neuroinflammation as well as Aβ peptides. Neuroinflammation is mainly mediated by microglial cells which are resident immune cells in the central nervous system (CNS) [6, 7]. Under physiologic conditions, microglial cells are involved in various functions, including the regulation of brain development, the maintenance of homeostasis, and the clearance of old synapses or other debris such as Aβ peptides. In AD, microglia has been known to be chronically activated, resulting in impairment of Aβ clearance, overexpression of pro-inflammatory signals, and consequently, neurotoxicity [8, 9]. Therefore, the regulation of microglial activation may be an important therapeutic strategy of AD by lowering excessive pro-inflammatory immune chemotaxis and enhancing neuroprotective function, leading to the modulation of neuroinflammation [10, 11].

Microglial activation plays an important role in several well-known pro-inflammatory signal cascades [12], including those mediated by mitogen-activated protein kinase (MAPK). The MAPK family is a family of serine/threonine protein kinases regulating cell properties in response to extracellular stimuli such as growth factors and inflammatory cytokines [13]. P38 MAPKs, one of the three MAPKs in mammalian cells, are primarily activated by inflammatory cytokines and environmental stresses [14]. P38 MAPKs have four isoforms α, β, γ, and δ which can be divided into two subgroups; one is p38α and p38β, and the other is p38γ and p38δ [15]. Among these, p38α and p38β MAPKs are highly expressed in adult mouse brain [16]. SB203580, a traditional p38α/β MAPK inhibitor, had shown therapeutic effects in the LPS-induced depression model and AD mouse model [17, 18]. Recently, two selective p38α MAPK inhibitors, neflamapimod (VX-745) and MW150, also showed therapeutic effects in aged rats and AD mouse model respectively [19, 20]. The phase 2a clinical trials of neflamapimod showed improvements in episodic memory in early AD patients [21, 22]. These results suggest that p38α/β MAPKs are potentially important targets in AD therapeutics, and thus, their inhibitors may be promising drugs.

In our previous study, a novel, selective p38α/β MAPK inhibitor, NJK14047, could successfully ameliorate microglia-mediated neuroinflammation [23]. It reduced inflammatory responses mediated by lipopolysaccharide (LPS) in the BV2 cells and the mouse model. Based on this finding, we intended to investigate the potential of NJK14047 as a therapeutic agent for AD. In this study, we used five familial Alzheimer’s disease (5XFAD) transgenic mice which have been commonly used as AD mouse models. 5XFAD mice overexpress human amyloid precursor protein (hAPP) with three FAD mutations [Swedish (K670N, M671L); Florida (I716V); and London (V717I)] and human presenilin 1 (PS1) with two FAD mutations (M146L and L286V) under the control of the neuron-specific *Thy1* promoter [24]. Here, the administration of NJK14047 to the 5XFAD mouse model was suggested to ameliorate memory loss, Aβ deposition, neuroinflammation, and neuronal degeneration via the selective inhibition of p38α/β MAPKs.

## Methods

### Chemicals and reagents

A selective p38α/β MAPK inhibitor NJK14047 was synthesized by a previously reported procedure (> 97%, HPLC) [23, 25]. Dulbecco’s modified Eagle’s medium (DMEM), fetal bovine serum (FBS), and penicillin/streptomycin were purchased from GE healthcare HyClone™. Cell culture flasks and plates were purchased from SPL (70075, 30006, 30024, 30048, 30096). LPS from *Escherichia coli* serotype O55:B5 (L6529, ≥ 500,000 EU/mg), methylthiazolyldiphenyl-tetrazolium bromide (MTT), thioflavin S (T1892), and DPX mounting medium (06522) were obtained from Sigma-Aldrich. Neurobasal medium (21103-049), B27 supplements (17504-044), RIPA buffer (89901), and protease/phosphatase inhibitor cocktail (78445) were obtained from ThermoFisher Scientific. The fluorescence-mounting medium (S3023) was purchased from Dako. TUNEL (G3250) assay kits were obtained from Promega. Information about antibodies used in this study is listed in Table 1.

**Table 1:** Information of immunostaining antibodies used in this study

Target	Host	Source	Catalog no.	RRID	Application
Phospho-p38 MAPK	Rabbit	Cell Signaling Technology	9215	AB_331762	WB/1:1000
p38 MAPK	Rabbit	Cell Signaling Technology	9212	AB_330713	WB/1:1000
Human β-amyloid 1–16 (clone: 6E10)	Mouse	BioLegend	803001	AB_2564653	WB/1:1000 IHC/1:500
BACE1	Mouse	Millipore	MAB5308	AB_11212616	WB/1:1000
Presenilin-1	Rabbit	Cell Signaling Technology	5643	AB_10706356	WB/1:1000
IDE	Rabbit	Abcam	ab32216	AB_775686	WB/1:1000
NEP	Goat	R&D System	AF1126	AB_2144426	WB/1:1000
Iba-1	Rabbit	Wako	019-19741	AB_839504	IHC/1:500
GFAP	Chicken	Abcam	ab4674	AB_304558	IHC/1:500
β-Actin (HRP)	Mouse	Santa Cruz Biotechnology	sc-47778 HRP	AB_2714189	WB/1:5000
Mouse IgG (HRP)	Goat	Santa Cruz Biotechnology	sc-2005	AB_631736	WB/1:5000
Rabbit IgG (HRP)	Goat	Santa Cruz Biotechnology	sc-2054	AB_631748	WB/1:5000
Goat IgG (HRP)	Donkey	Santa Cruz Biotechnology	sc-2020	AB_631728	WB/1:5000
Mouse IgG (488)	Goat	Invitrogen	A11001	AB_2534069	IHC/1:1000
Rabbit IgG (488)	Goat	Invitrogen	A11008	AB_143165	IHC/1:1000
Rabbit IgG (594)	Goat	Invitrogen	A11012	AB_2534079	IHC/1:1000
Chicken IgY (488)	Goat	Invitrogen	A11039	AB_2534096	IHC/1:1000

*WB* Western blot, *IHC* immunohistochemistry

### Animals and treatment

5XFAD mice on B6/SJL background (34840-JAX, Tg6799) and wild-type B6/SJL mice from Jackson Laboratory were bred and maintained in an individual ventilated cage with 12-h light/dark cycles at 22 °C. 5XFAD mice were divided into two experimental groups (treatment vs. vehicle group) using the block randomization method. Similar to previous studies [26, 27], the treatment group was treated with NJK14047 at 2.5 mg/kg every other day from the age of 6 to 9 months by intraperitoneal injection. The vehicle group and its littermate wild-type mice were treated with the same volume of vehicle by intraperitoneal injection. NJK14047 was dissolved in pure DMSO at 25 mg/ml which was used as a 40X stock solution. For working solution, an aliquot of the 40X stock solution was diluted in PBS and filtered using a 0.25-μm syringe filter. The body weights of the mice did not significantly differ among groups, and any conspicuous side effects (weight loss, anorexia, convulsion, or death) were not observed. All mice used in this study were sacrificed at 9 months of age. Both sexes of mice were used in Morris water maze test and Aβ_1–42_ ELISA without any effect of sex on therapeutic effects. To minimize the sex difference on the degree of amyloidopathy, the protein and RNA samples were obtained from male mice, and histological sections from female mice. All experiments were approved by the Kyung Hee University Institutional Animal Care and Use Committee (IACUC, KHUASP(SE)-17-126-1).

### Behavioral test

The Morris water maze task was used to assess spatial memory performance. The water maze was a white circular PVC tank (130-cm diameter, 40-cm height) in which water was filled to 31.5 cm in depth (23 ± 1 °C). The submerged target platform was a 10-cm diameter circular zone located 1.5 cm below the surface of the water. Titanium dioxide (TiO_2_) was dispersed in water to camouflage the target platform. The position of the platform was varied for each mouse with equal distribution between experimental groups. One day before training, mice were habituated to the maze and swimming in the absence of cues. During the training period, mice were subjected to four trials per day, and the start position of each trial was equally distributed. In each trial, the mouse was given 60 s to find the target platform in the presence of cues around the maze. The time spent finding the platform was recorded as the latency of each trial. If a mouse did not find the platform within 60 s, it was guided to the platform and allowed to stay on the platform for 10 s. After a total 10 days of training, a probe test was performed (day 11). The mouse was subjected to a trial in the absence of the platform for 60 s. The start position was standardized to the opposite direction of the area where the platform was located. All trials were recorded using a camera and analyzed by a free tracking software tool, Toxtrac [28]. The software, the user manual, and the documentation are available at https://toxtrac.sourceforge.io.

### Immunoblotting

Immunoblotting analysis was performed as previously described [29]. In brief, mice were sacrificed after the behavioral test. The cortex and hippocampus were quickly isolated and stored at − 80 °C until use. The brain tissues were homogenized in 10X volume of lysis buffer (RIPA buffer containing 1% 100X protease inhibitor) and centrifuged at 13,000 rpm for 20 min. The supernatant was collected, and the concentrations were measured using the Bradford method (Bio-Rad, 5000006). Equal amounts of protein samples (~ 50 μg) were fractionated by SDS-PAGE and then transferred to PVDF membranes. The membranes were blocked with 5% skim milk and probed with primary antibodies at 4 °C overnight. After several washes in TBS-T, the membranes were probed with corresponding secondary antibodies conjugated with HRP. The immunoblot signals were developed with an enhanced chemiluminescence detection system (ECL, Bio-Rad, 1705061). The band intensity was quantified using ImageJ densitometry (NIH, Bethesda, MD, USA), normalized with respect to the β-actin level.

### Tissue preparation and immunofluorescence

The mice were anesthetized with intraperitoneal injection of 2.5% Avertin (2,2,2-tribromoethanol) and immediately perfused through the heart with PBS followed by 4% paraformaldehyde in PBS. Brains were excised, post-fixed in 4% paraformaldehyde at 4 °C overnight, and incubated in 30% sucrose at 4 °C until reaching equilibrium. The brains were embedded in O.C.T. compound blocks at − 80 °C. Sequential 30-μm coronal sections were obtained with a cryostat (CM30 50S; Leica). Every tenth section (300 μm apart) of the brain (Bregma − 1.30 to − 2.70 mm) was used for immunohistochemistry. Free-floating brain sections were rinsed in PBS; blocked for 1 h in 2% normal goat serum, 2% BSA, and 0.4% Triton-X100; and then incubated with primary antibodies at 4 °C overnight. After incubation with primary antibodies, the brain sections were washed with PBS and incubated for 2 h with corresponding secondary antibodies conjugated with Alexa fluorescence.

### Thioflavin S staining

To stain fibrillary Aβ, the brain sections were incubated with 0.1% thioflavin S in 50% EtOH for 10 min. Brain sections were sequentially washed with 50% EtOH, 70% EtOH, and 100% EtOH for 5 min, respectively; afterward, a coverslip was applied over the fluorescence-mounting medium.

### Aβ ELISA

An enzyme-linked immunosorbent assay (ELISA) for human Aβ_1–42_ was performed using fluorescence-based ELISA kits (Invitrogen) and appropriate Aβ standards in compliance with the manufacturer’s protocol. The hippocampus and frontal cortex from one hemisphere were homogenized in 10X volume of guanidine buffer with a final concentration of 50 mM Tris and 5 M guanidine HCl at pH 8.0. Homogenates were mixed at room temperature for 4 h and then diluted in PBS containing 5% BSA, 0.03% Tween 20, and protease inhibitor cocktail.

### Quantitative real-time polymerase chain reaction (qRT-PCR)

Total RNA was extracted from the mouse cortex and hippocampus with a Hybrid-R total RNA purification kit (GeneAll®, 305-101) in accordance with the manufacturer’s instructions. The NanoDrop™-2000c (ThermoFisher Scientific) was used to assess the concentration and purity of the RNA samples. cDNA was synthesized using TOPscript RT DryMIX (Enzynomics, RT200), according to the manufacturer’s instructions. cDNA samples were subjected to qRT-PCR using SYBR Green Mix (Enzynomics, RT500) and a CFX Connect real-time PCR system (Bio-Rad). The qRT-PCR protocol was as follows: first holding stage at 95 °C for 3 min; followed by a cycling stage at 95 °C for 10 s, 55 °C for 10 s, 72 °C for 30 s (30 cycles total); and finally, a holding stage at 95 °C for 10 s. The information about the primers used in this study is listed in Table 2. The measured data were normalized to the GAPDH level using the 2^-ΔΔCT^ method and expressed as the fold change with respect to the mean of the control group.

**Table 2:** Information of qRT-PCR primers used in this study

Gene name	NCBI reference	Forward primer (5′ → 3′)	Reverse primer (5′ → 3′)
TNF-α	NM_013693	GATTATGGCTCAGGGTCCAA	GCTCCAGTGAATTCGGAAAG
IL-1β	NM_008361	CCCAAGCAATACCCAAAGAA	GCTTGTGCTCTGCTTGTGAG
IL-6	NM_031168	CCGGAGAGGAGACTTCACAG	TTGCCATTGCACAACTCTTT
Arg1	NM_007482	AGTTGGGTTCACTTCCATGA	CGATTCACCTGAGCTTTGAT
YM-1	NM_009892	AGAGCAAGAAACAAGCATGG	CTGTACCAGCTGGGAAGAAA
Fizz	NM_020509	TCCAGCTAACTATCCCTCCACTGT	GGCCCATCTGTTCATAGT
MACRO	NM_010766	CTGTGGCAATGGATCACTAGC	CTCCTGGCTGGTATGGACC
Msr1	NM_031195	TGAACGAGAGGATGCTGACTG	GGAGGGGCCATTTTTAGTGC
SCARB1	NM_016741	TTTGGAGTGGTAGTAAAAAGGGC	TGACATCAGGGACTCAGAGTAG
SCARB3	NM_007643	GAACCACTGCTTTCAAAAACTGG	TGCTGTTCTTTGCCACGTCA
GAPDH	NM_008084	TGAATACGGCTACAGCAACA	AGGCCCCTCCTGTTATTATG

### Fluoro-Jade B staining

To detect late-stage degenerating neuronal cells, Fluoro-Jade B staining was performed [30, 31]. Brain sections were incubated with 1% NaOH in 80% EtOH for 5 min, followed by sequential washes with 70% EtOH and distilled water. Afterward, the brain sections were incubated with 0.06% potassium permanganate for 10 min, followed by a 2-min wash with distilled water. The sections were then incubated in 0.0004% Fluoro-Jade B (Millipore, AG310) plus 0.01% acetic acid solution. After three washes with distilled water, the sections were fully dried and coverslipped with the DPX mounting medium.

### Cell lines and treatment

The BV2 (RRID: CVCL_0182) murine microglia cell line and the C8-D1A (RRID: CVCL_6379) murine astrocyte cell line were cultured in DMEM with 10% FBS, 100 U/ml penicillin, and 100 μg/ml streptomycin at 37 °C in a humidified atmosphere of 5% CO_2_. Cells were seeded on 6-well plates at 5 × 10^5^ cells/well and stimulated with 500 ng/ml LPS after 2 h of pre-treatment with either 1 μM or 10 μM NJK14047. After 22 h of LPS stimulation, all media were changed to fresh neurobasal medium. The cells were incubated in neurobasal media for another 24 h. The conditioned neurobasal medium was obtained and centrifuged at 1500 rpm for 5 min to remove the remaining cells. The supernatant was collected and stored at 4 °C and then used to culture primary neurons within 24 h.

### Primary cells and treatment

Primary mouse cortical microglia and astrocytes were prepared according to the commonly used protocols as previously described [32, 33]. Briefly, mouse cortical mixed glia was obtained from P1 to P4 C57BL/6J mouse pups and seeded on poly-l-lysine pre-coated 75T flasks at 1.5 × 10^7^ cells/flask. The culture medium was changed every 3 days. After 5 to 7 days, the flasks were vigorously tapped, and the supernatant was collected and centrifuged at 3000 rpm for 10 min to obtain microglia. The cell pellet was dispersed in a new medium and seeded on poly-l-lysine pre-coated 6-well plates at 5 × 10^5^ cells/well. After 2 h of cell seeding, the culture medium was replaced with a fresh medium to remove the oligodendrocytes. Approximately 96% of the cells were found to be positive for ionized calcium-binding adapter molecule 1 (Iba-1) which is a marker of microglia. Afterward, the flasks were tapped, and the supernatant was discarded; the rest of the cells in the flask were rinsed with PBS and seeded onto poly-l-lysine pre-coated 6-well plates at 5 × 10^5^ cells/well to harvest astrocytes. Approximately 98% of the cells were positive for glial fibrillary acidic protein (GFAP) which is a marker of astrocytes. Prepared microglia and astrocytes were stimulated with 50 ng/ml LPS after 2 h of pre-treatment with 10 μM NJK14047. After 22 h of LPS stimulation, all media were changed to a fresh neurobasal medium to incubate the cells for another 24 h. The conditioned neurobasal medium was obtained and centrifuged at 1500 rpm for 5 min. The supernatant was collected, stored at 4 °C, and then treated to primary neurons within 24 h.

Primary mouse cortical neurons were prepared as previously described [34]. In brief, mouse cortical neurons were obtained from E17 C56BL/6J mouse embryos. The neurons were seeded on poly-l-lysine pre-coated 48-well plates or 24-well coverslips for the MTT and the TUNEL assay, respectively. The neurobasal medium containing 2% B27 supplement, 2 mM l-glutamine (Welgene, LS 002-01), and 1% P/S was used to culture neurons. Primary neurons were used in experiments at 10 days in vitro (DIV).

### Cytotoxicity assay

The MTT assay is generally used to assess the cell viability by measuring the metabolic activity of the cells [35]. To assess the cytotoxicity of the glia-conditioned medium, mouse cortical primary neurons were seeded on poly-l-lysine pre-coated 48-well plates at 1 × 10^5^ cells/well. On DIV10, neurons were incubated in the medium conditioned with BV2, C8-D1A, primary cultured microglia, and primary cultured astrocytes, respectively, for 24 h. The media were replaced with neurobasal media containing 10% MTT. Absorbance at 570 nm corrected with 690-nm values was measured by a microplate reader. The measured cell viability was expressed as a percentage relative to the control mean.

### TUNEL assay

Apoptotic DNA fragmentation was detected using the TUNEL method [36]. To measure the apoptotic cell death of primary neuronal cells, mouse cortical primary neurons were seeded on poly-l-lysine pre-coated 24-well cover glasses at 1.5 × 10^5^ cells/well. On DIV10, neurons were incubated in each conditioned medium for 24 h. After incubation, the cells were subjected to TUNEL staining in accordance with the manufacturer’s instructions. Apoptotic cells were detected as localized bright green cells (positive cells) in a blue background using an Olympus BX51 microscope. The extent of apoptosis was quantified using ImageJ software.

### Confocal microscopy and image analysis

All stained brain sections were imaged by confocal microscopy. Z-stacked images were acquired at 1.5-μm intervals (total 15 optical slices). Four cortex areas (2 left; 2 right) and four hippocampus areas (2 left; 2 right) were imaged in one brain slice. For each mouse, thioflavin S-positive area, 6E10-positive area, and Fluoro-Jade B-positive cells were quantified with four to five brain slices (Bregma − 1.30 to − 2.70, 300 μm apart) using ImageJ software. The number of Iba-1- and GFAP-positive cells were quantified using Cell Profiler software [37]. To quantify the number of plaque-associated microglia, Iba-1-positive cells within the 20-μm range of plaques were manually counted. A total of 70–75 plaques (> 10 μm) from five mice per group were measured [26].

### Statistical analysis

In all in vivo studies, *n* represents the number of animals used in the corresponding experiment. For in vitro studies, *n* is the number of independent experiments. The group size for each experiment was based on our previous results [38]. The operators responsible for the experimental procedure and data analysis were blinded and unaware of group allocation throughout the experiments. All data were analyzed using SPSS version 25 (IBM corporation, NY, USA) with statistical significance defined as a *P* value less than 0.05. Parametric tests such as ANOVA were used when the data satisfied the null hypothesis of the Levene’s test. Tukey’s post hoc test was performed if the *P* value was < 0.05 in one-way ANOVA. In case of qRT-PCR and the MTT assay, the Kruskal-Wallis test followed by Dunn’s post hoc multiple comparisons was used to compare among the experimental groups. Latency of Morris water maze task was analyzed using the generalized estimating equation (GEE) method. Descriptive statistics were used to summarize the data using mean ± standard error of the mean (SEM). All graphs were constructed using GraphPad Prism 5.0 software (GraphPad software Inc., CA, USA).

## Results

### NJK14047 as a selective p38α/β MAPK inhibitor reduces the level of phospho-p38 MAPKs in the brain and attenuates spatial memory loss in 9-month-old 5XFAD mice

**Fig. 1:**
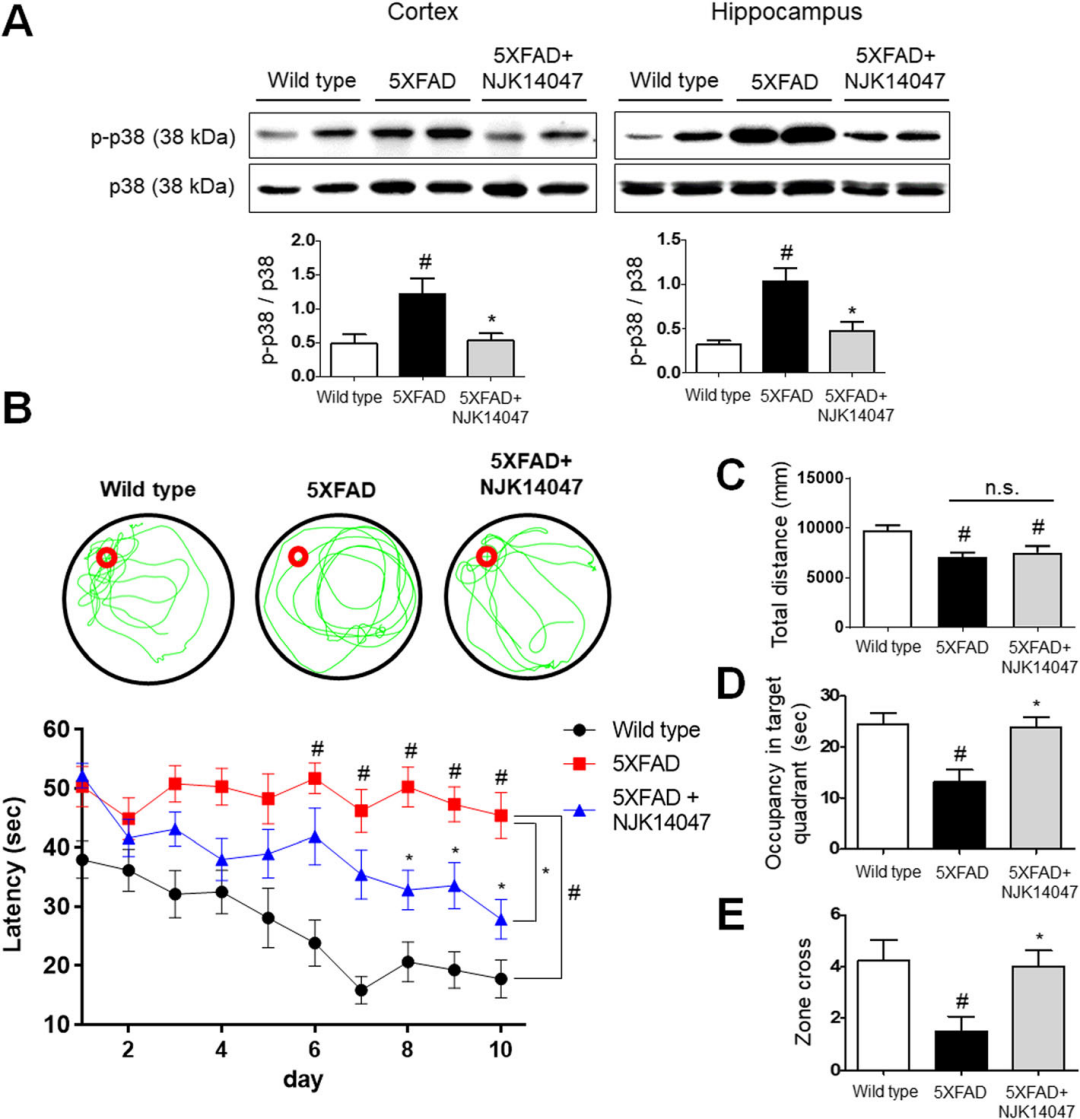
NJK14047 treatment from 6 to 9 months of age reduces phospho-p38 MAPK level and prevents spatial learning memory impairment. **a** Inhibition of p38 MAPK in the cortex and hippocampus of NJK14047-treated 9-month-old 5XFAD mice. Phospho-p38 protein level was normalized to the total p38 level and shown as mean ± SEM (*n* = 6 per group, one-way ANOVA). **b** Morris water maze task between three groups: wild-type, 5XFAD, and 5XFAD + NJK14047. Representative swimming paths during a probe task after 10 days of training and escape latencies of each group for total 10 days of training. Data are shown as mean ± SEM (*n* = 12–13 per group, GEE analysis). **c**–**e** After 10 days of training, a probe task was performed for the mice. **c** Total swimming distance was measured without significant difference between the 5XFAD and 5XFAD + NJK14047 group. **d** Time spent in the target quadrant and **e** the frequency of crossing the target zone during a 60-s probe test. Data are shown as mean ± SEM (*n* = 12–13 per group, one-way ANOVA). ^#^*P* < 0.05 vs. wild type; *P < 0.05 vs. 5XFAD

The phospho-p38 MAPK level was upregulated in the brain of 9-month-old 5XFAD mice in both cortex and hippocampus regions of the brain compared to the wild-type mice (Fig. 1a). The upregulated phospho-p38 MAPK levels were significantly reduced in the NJK14047-treated 5XFAD mouse brain to the level comparable to that of the WT mice. This inhibitory effect was observed in both cortex and hippocampus regions (Fig. 1a).

Based on the suppressive effects of NJK14047 on the p38 MAPKs pathway in 5XFAD mice, we further evaluated whether NJK14047 treatment could ameliorate spatial learning memory impairment in 5XFAD mice. NJK14047- and vehicle-treated 5XFAD mice as well as their wild-type littermates were subjected to Morris water maze task [39]. As shown in Fig. 1b, overall, 5XFAD mice showed markedly longer escape latency than wild-type mice (*P* < 0.05 since day 6). However, the escape latency was significantly reduced with NJK14047 treatment compared to the control 5XFAD mice (*P* < 0.05 since day 8) (Fig. 1b). At day 11, a probe task was performed with the hidden platform removed. Representative swimming paths of each group during the probe task were shown in Fig. 1b. 5XFAD mice showed less total swimming distance compared to the wild-type littermates without significant difference between NJK14047- and vehicle-treated groups (Fig. 1c). The recorded time spent in the target quadrant was 24.48 ± 2.14 s in wild-type mice and 13.14 ± 2.35 s in 5XFAD mice (*P* < 0.01). The elapsed time in the target quadrant was significantly increased in the NJK14047-treated 5XFAD mice (23.79 ± 2.08 s, *P* < 0.01 vs. 5XFAD mice without NJK14047 treatment) (Fig. 1d), which was comparable to that in the wild-type mice. Target zone cross also differed between groups in a 60-s probe task. The wild-type group and the 5XFAD + NJK14047 group showed 4.25 ± 0.77 times and 4.00 ± 0.62 times, respectively, whereas the frequency of crossing the target zone was significantly lower in the 5XFAD group without treatment (1.50 ± 0.57 times, *P* < 0.05) (Fig. 1e). These results suggest NJK14047 treatment reduces phospho-p38 MAPK levels in the 5XFAD mouse brains and ameliorates spatial learning memory loss in 9-month-old 5XFAD mice.

### NJK14047 decreases Aβ deposits in the brain of 9-month-old 5XFAD mice

**Fig. 2:**
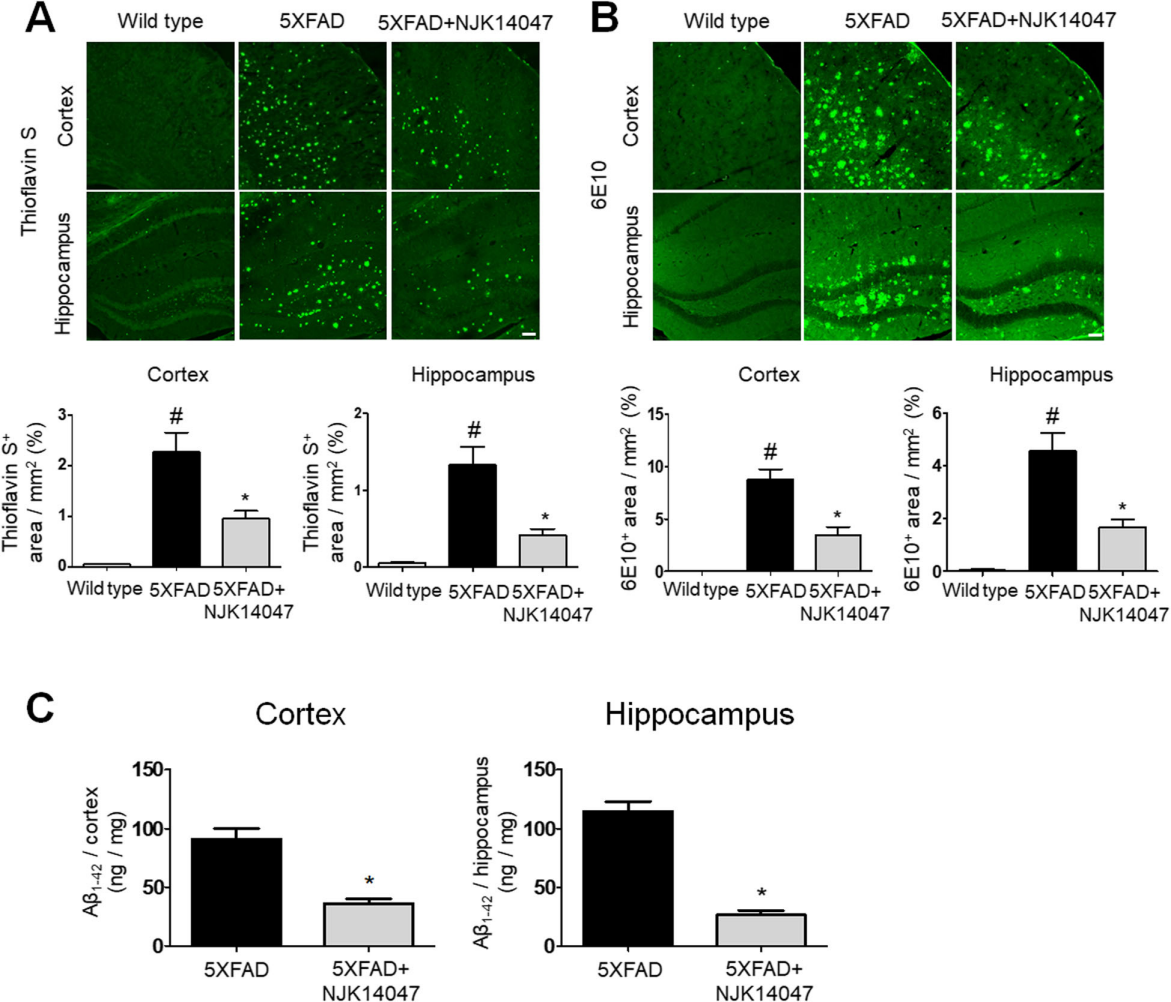
NJK14047 treatment from 6 to 9 months of age reduces Aβ plaques in the cortex and hippocampus. **a**, **b** Representative confocal microscope images of mouse brain slices stained with **a** thioflavin S dye and **b** 6E10 antibody. Thioflavin S- and 6E10-positive areas per square millimeter were quantified. For quantification, four areas in each section and four sections in each mouse were used. Data are shown as mean ± SEM (scale bar = 100 μm, *n* = 5 per group, one-way ANOVA). **c** Aβ protein level per brain cortex and hippocampus weight (mg) was measured using Aβ_1–42_ ELISA. Data are shown as mean ± SEM (*n* = 6 per group, Student’s *t* test). ^#^*P* < 0.05 vs. wild type; *P < 0.05 vs. 5XFAD

As a next step, we investigated whether Aβ deposits, one of the representative hallmarks in AD, were altered with NJK14047 treatment in the 5XFAD mice. As shown in Fig. 2a, thioflavin S-positive Aβ plaques were observed in 9-month-old 5XFAD mice, while no plaque was detected in their wild-type littermates. Notably, the thioflavin S-positive area was decreased in both cortex and hippocampus regions of NJK14047-treated 5XFAD mice. Immunostaining with a 6E10 antibody against human Aβ_1–16_ showed similar results, supporting the reduction of Aβ plaques by NJK14047 treatment in the brain (Fig. 2b). This Aβ reduction as a result of NJK14047 administration was further confirmed using human Aβ_1–42_ ELISA kit (Fig. 2c). According to these data, NJK14047 treatment attenuates Aβ deposits in the 9-month-old 5XFAD mouse brain.

### Aβ processing and degradation are not affected by NJK14047

**Fig. 3:**
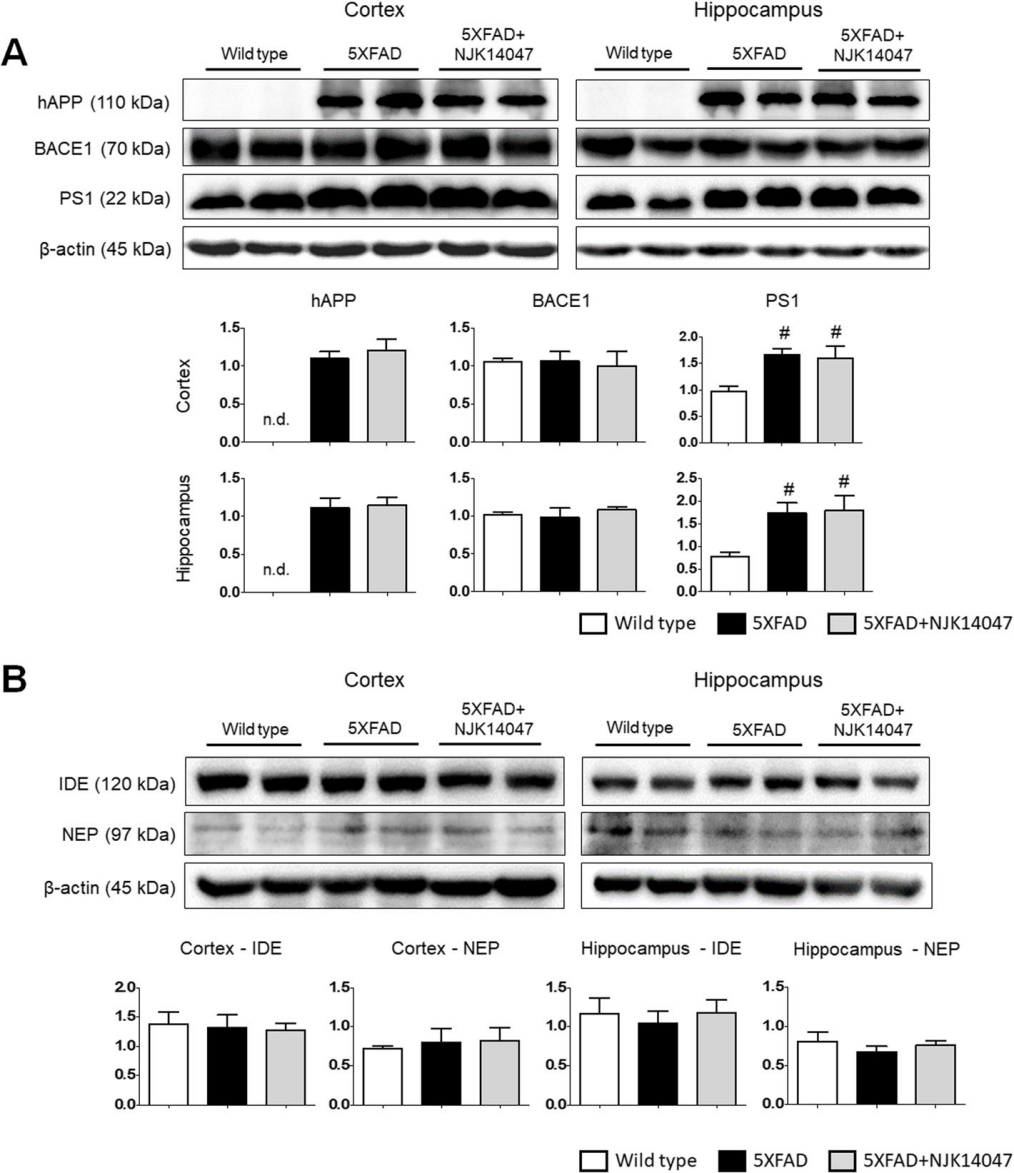
NJK14047 treatment from 6 to 9 months of age does not affect amyloid beta precursor protein processing and degrading molecules. **a** Representative immunoblotting images and quantifications for proteins involved in the production of Aβ. Total protein lysates of the cortex and hippocampus were used and immunoblotted with 6E10, BACE1, and PS1 antibodies. Protein levels were quantified using ImageJ, normalized to its β-actin level. Data were shown as mean ± SEM (*n* = 5 per group, one-way ANOVA). **b** Representative immunoblotting images and quantifications for proteins involved in the elimination of Aβ. Immunoblotting was performed with IDE and NEP antibodies using total protein lysates of the cortex and hippocampus. Quantitative analysis was performed using ImageJ. The quantified protein levels were normalized to the β-actin level and expressed as mean ± SEM (*n* = 5 per group, one-way ANOVA)

Reduction of Aβ plaques in the NJK14047-treated mouse brain might result from the decrease in the APP production or the reduction in the level of proteins related to Aβ processing or degradation. To investigate this hypothesis, the levels of hAPP, BACE1, and PS1 proteins were examined by immunoblotting. No significant change in hAPP, BACE1, and PS1 levels was observed in NJK14047-treated mouse brains compared to those in 5XFAD mouse brains, suggesting the decrease in Aβ plaques by NJK14047 does not result from the reduction in APP processing (Fig. 3a). In addition to APP processing molecules, proteins involved in Aβ degradation, such as insulin degrading enzyme (IDE) and neutral endopeptidase (neprilysin, NEP), could also regulate Aβ deposits [40, 41]. Similar to APP processing molecules, the protein levels of Aβ degrading enzymes were not significantly different among the experimental groups (Fig. 3b). These results suggest NJK14047 treatment could not directly alter the APP processing pathway or Aβ degrading enzymes in 9-month-old 5XFAD mice.

### NJK14047 inhibits neuroinflammatory conditions in the brains of 9-month-old 5XFAD mice

**Fig. 4:**
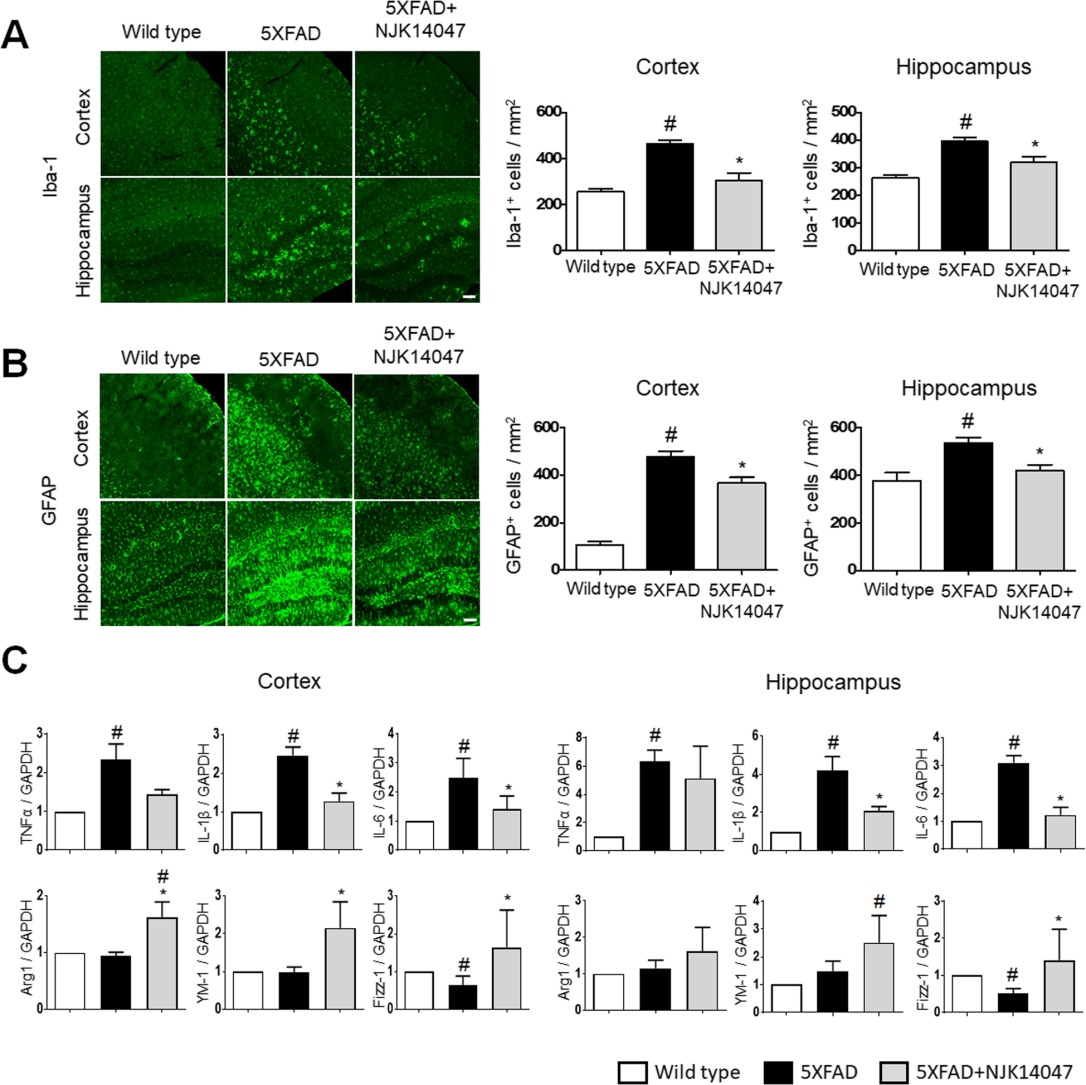
NJK14047 treatment from 6 to 9 months of age downregulates neuroinflammation in the cortex and hippocampus. **a**, **b** Representative confocal microscope images of mouse brain sections stained with **a** Iba-1 antibody for labeling microglia and **b** GFAP antibody for labeling astrocytes. Iba-1- and GFAP-positive cell counts per square millimeter were measured. Data are shown as mean ± SEM (scale bar = 100 μm, *n* = 5 per group, one-way ANOVA). **c** Relative mRNA expression levels of M1 and M2 microglial markers were measured using qRT-PCR. Total mRNA was extracted in the mouse cortex and hippocampus. The mRNA expression levels were normalized to GAPDH and represented as the fold change with respect to the mean of the control group with SD (*n* = 5–6 per group, Kruskal-Wallis test). ^#^*P* < 0.05 vs. wild type; *P < 0.05 vs. 5XFAD

Because neuroinflammation has been suggested to contribute to AD pathogenesis and is related to Aβ production and elimination [6, 42], we examined the neuroinflammation state in our 5XFAD mice. First, we assessed the gliosis of microglia and astrocytes in 5XFAD mice. As expected, 9-month-old 5XFAD mice exhibited higher extent of microgliosis than wild-type littermates in the cortex and hippocampus. This upregulated microgliosis was significantly decreased in NJK14047-treated 5XFAD mice (Fig. 4a). Similar to microgliosis, increased astrogliosis was decreased in the NJK14047-treated 5XFAD group (Fig. 4b). For further investigation, the microglial activation markers were analyzed using qRT-PCR. Consistent with the gliosis results, the expression levels of pro-inflammatory cytokines such as tumor necrosis factor α (TNF-α), interleukin 1β (IL-1β), and interleukin 6 (IL-6) were significantly elevated in the 5XFAD mouse brains compared to those in the wild-type brains (Fig. 4c, upper). On the other hand, the levels of arginase 1 (Arg1), chil3 chitinase-like 3 (Chi3l3/YM-1), and resistin-like alpha (Retnla/Fizz-1), which indicate alternative active state of microglia, were not significantly different between the wild-type and 5XFAD mice except Fizz-1 (Fig. 4c, lower). The expression levels of pro-inflammatory cytokines were significantly decreased in the NJK14047-treated 5XFAD mouse brains except TNF-α (Fig. 4c, upper). On the contrary, Arg1, YM-1, and Fizz-1 were upregulated in the brain cortex of NJK14047-treated 5XFAD mice. In the hippocampus, Arg1 and YM-1 expression levels were apparently increased in the 5XFAD + NJK14047 group without reaching statistical significance; for Fizz-1, the expression level was significantly increased compared to the 5XFAD group (Fig. 4c, lower). Taken together, these results indicate NJK14047 treatment alleviates inflammatory conditions in the 9-month-old 5XFAD mouse brain.

### NJK14047 increases phagocytic activity of microglia in association with Aβ clearance

**Fig. 5:**
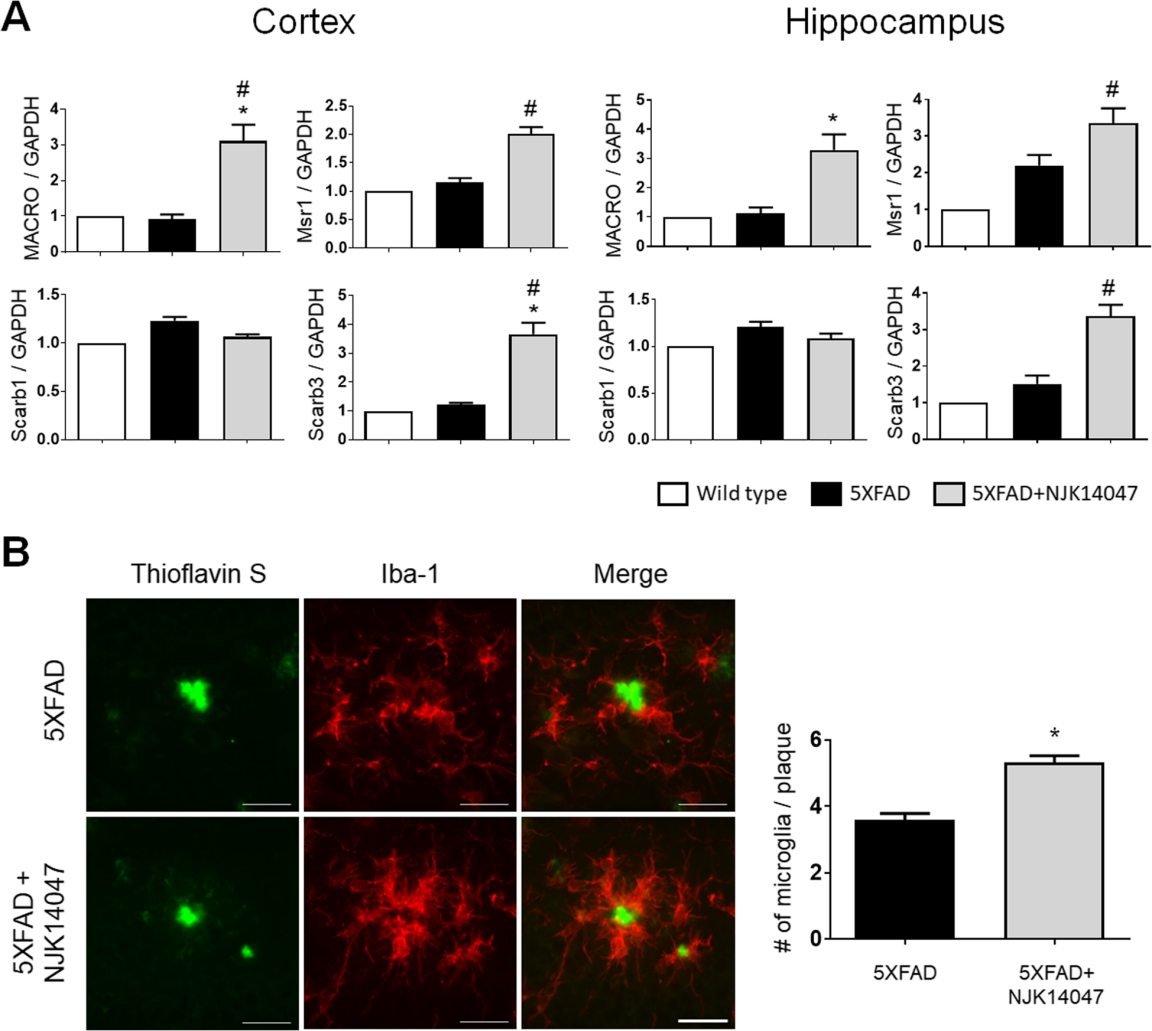
NJK14047 treatment from 6 to 9 months of age upregulates the expression levels of microglial phagocytic receptors and the Aβ clearance function of microglia. **a** Relative mRNA expression levels of microglial phagocytic receptors in the cortex and hippocampus were measured using qRT-PCR. The expression levels were normalized to GAPDH levels and shown as the fold change with respect to the mean of the control group with SD (*n* = 5–6 per group, Kruskal-Wallis test). **b** Representative confocal microscope images for phagocytic microglia stained with thioflavin S and Iba-1 antibody (scale bar = 30 μm). Microglial cell counts within the 20-μm range of Aβ plaque were quantified. Data were shown as mean ± SEM (*n* = 5 per group, Student’s *t* test). ^#^*P* < 0.05 vs. wild type; *P < 0.05 vs. 5XFAD

The alternative activation of microglia was previously shown to be effective for phagocytosis and degradation of Aβ peptides [43]. To further confirm the alleviation of the neuroinflammatory condition in 5XFAD mice as a result of NJK14047 treatment, we investigated the phagocytic function of microglia in 9-month-old 5XFAD mice. The mRNA expression levels of the following phagocytic receptors in the brain were measured using qRT-PCR: macrophage receptor with collagenous structure (MACRO); macrophage scavenger receptor 1 (Msr1); scavenger receptor class B, member 1 (Scarb1); and scavenger receptor class B, member 3 (Scarb3). As expected, NJK14047-treated 5XFAD mice showed increased expression levels of phagocytic receptors compared to wild-type or vehicle-treated 5XFAD mice except Scarb1 (Fig. 5a). For histological examination, the brain sections were co-stained with thioflavin S and Iba-1 antibody. Consistent with qRT-PCR data, microglia were primarily found to cluster around Aβ plaques in the NJK14047-treated mouse brains (Fig. 5b). These data indicate NJK14047 treatment induces anti-inflammatory responses and improves microglial phagocytosis of Aβ peptides in 9-month-old 5XFAD mice.

### NJK14047 reduces the neuronal cell death in the cortex and hippocampus

**Fig. 6:**
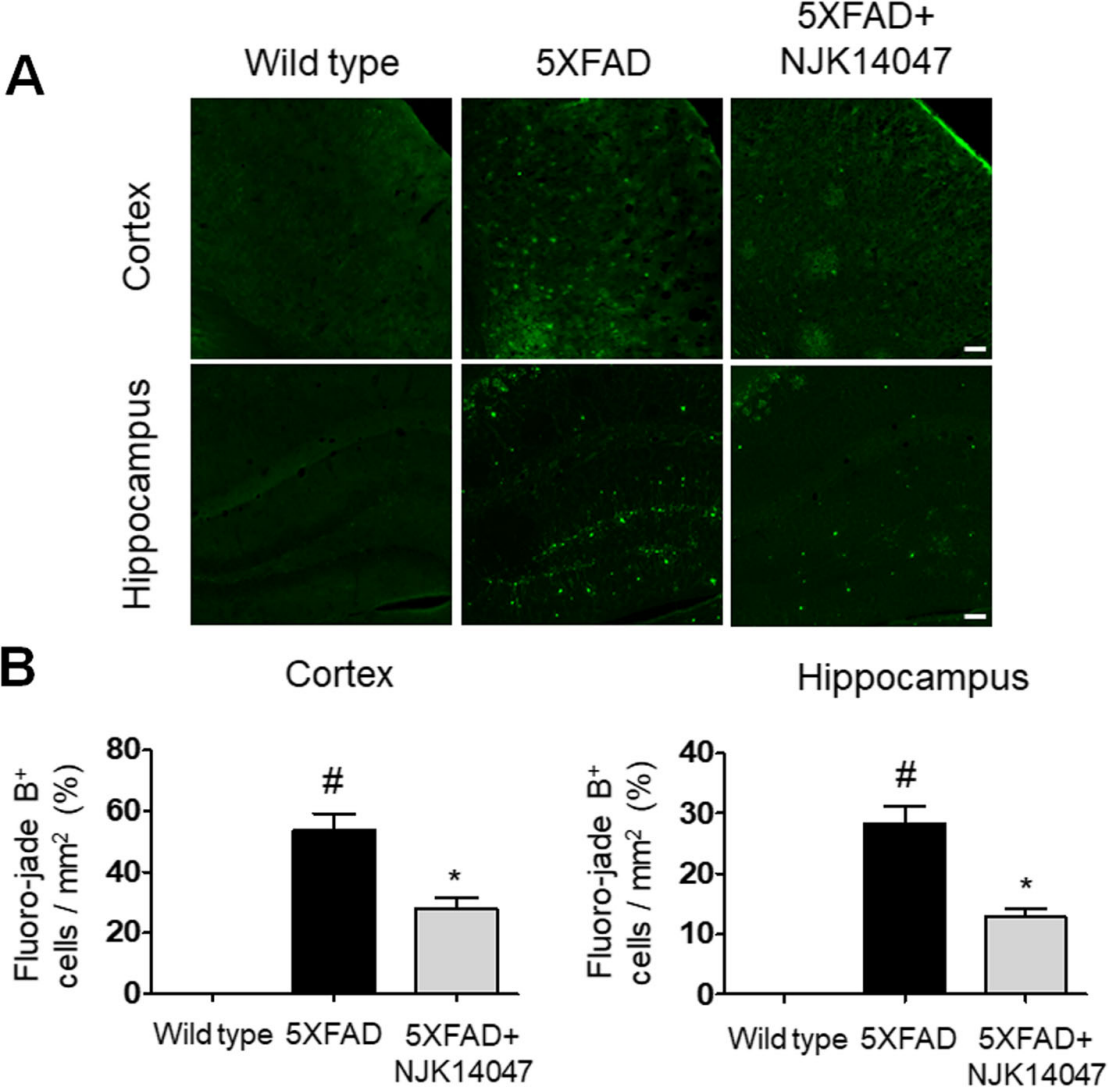
NJK14047 treatment from 6 to 9 months of age inhibits neuronal deaths in the cortex and hippocampus. **a** Representative confocal microscope images of mouse brain sections stained with Fluoro-Jade B dye for labeling degenerating neurons in the cortex and hippocampus (scale bar = 100 μm). **b** Quantitative analysis was performed with four sections in each mouse and four areas in each section. Fluoro-Jade B-positive cells per square millimeter were quantitated. Data are shown as mean ± SEM (*n* = 5 per group, one-way ANOVA). ^#^*P* < 0.05 vs. wild type; *P < 0.05 vs. 5XFAD

According to previous studies, Aβ deposits and chronic neuroinflammation may provoke neuronal cell death, contributing to important pathology in AD [44–47]. In this present study, Aβ plaques and neuroinflammation were substantially decreased in NJK14047-treated 9-month-old 5XFAD mice; therefore, we tried to confirm our hypothesis that the p38 MAPK inhibitor might reduce the neuronal cell death in 5XFAD mice. As expected, we observed Fluoro-Jade B-positive cells in the cortex and hippocampus of 9-month-old 5XFAD mice, but not in wild-type mice. Pre-treatment with NJK14047 in the 5XFAD mice led to fewer Fluoro-Jade B-positive cells in both brain regions compared to those without pre-treatment (Fig. 6). This finding is consistent with our speculation that neuronal cell death could be decreased in association with the reduction in Aβ plaques as well as alleviation of neuroinflammatory state in the 5XFAD mouse brains, which might result from the pharmacological inhibition of p38α/β MAPKs.

### NJK14047 decreases neurotoxicity mediated by the activated microglia

**Fig. 7:**
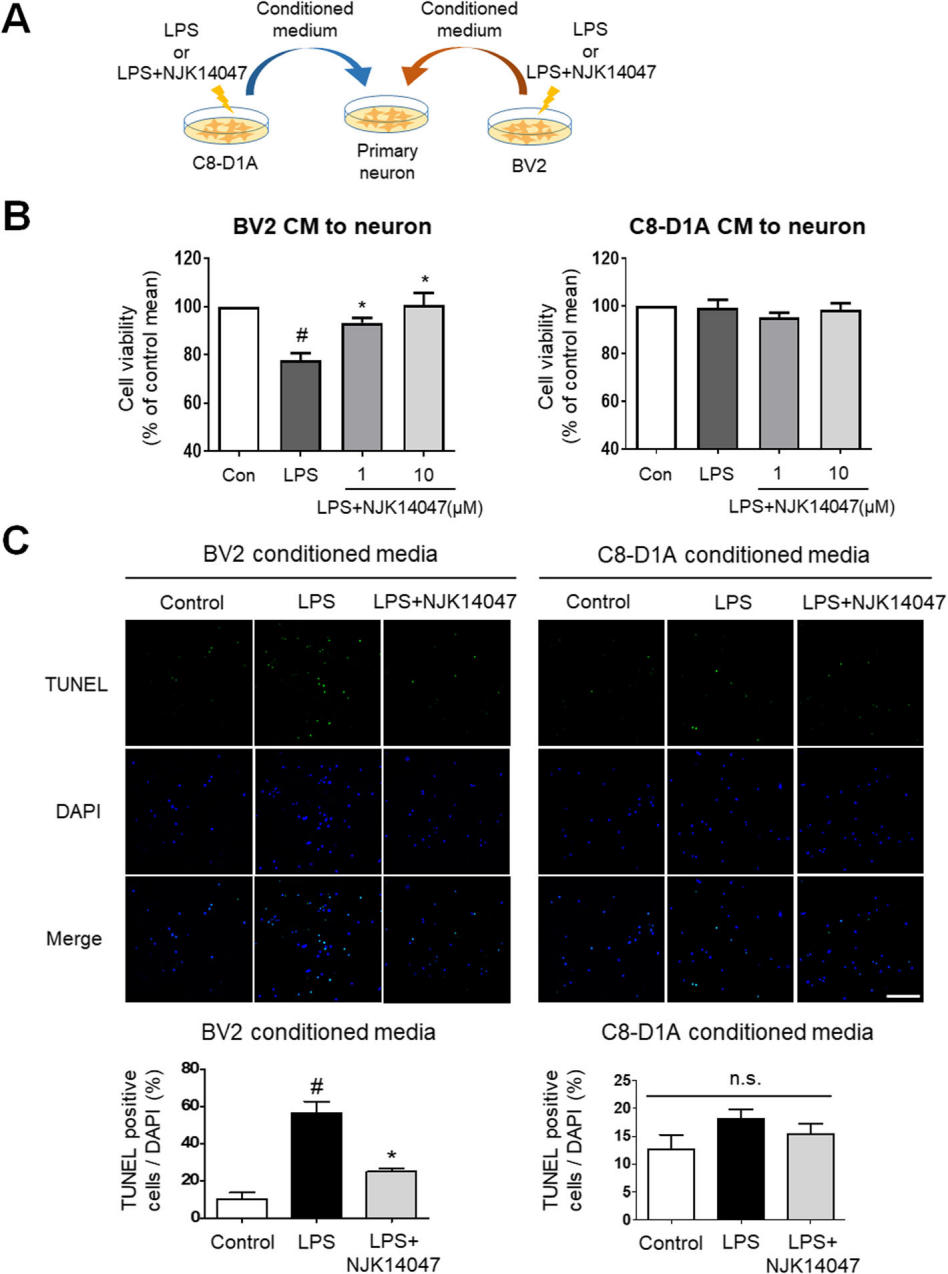
NJK14047 attenuates neurotoxicity of LPS-stimulated microglial cell line rather than that of LPS-stimulated astrocyte cell line. **a** Scheme of the experiment. Microglial cell line BV2 and astrocyte cell line C8-D1A were seeded on 6-well plate at 5 × 10^5^ cells/well. BV2 and C8-D1A were stimulated with 500 ng/ml LPS alone or after 2 h of pre-treatment with either 1 or 10 μM NJK14047. After 22 h of LPS stimulation, all media were changed to the fresh neurobasal medium where cells were incubated for 24 h. The conditioned medium was collected to treat primary neurons seeded on 48-well plate for cytotoxicity assay (**b**) or 24-well cover glass for TUNEL assay (**c**). **b** Viability of mouse primary neurons stimulated with BV2 and C8-D1A conditioned media. O.D. at 570 nm were normalized to the control group and shown as percentage of control mean ± SD (*n* = 3, Kruskal-Wallis test). **c** TUNEL assay for mouse primary neurons stimulated with BV2 and C8-D1A conditioned media. Apoptotic cells were quantified using four images in each cover glass. Data are shown as mean ± SEM (scale bar = 100 μm, *n* = 5, one-way ANOVA). ^#^*P* < 0.05 vs. the control group; *P < 0.05 vs. the LPS group

To delineate the relations between neuroinflammation suppression by NJK14047 and the neuroprotective effect in AD mice, the potential pharmacological effects of NJK14047 were evaluated using primary neurons, microglial cell line BV2, and astrocyte cell line C8-D1A. BV2 and C8-D1A were cultured in vitro, and the conditioned media (CM) of BV2 and C8-D1A were prepared as described in the “Methods” section. The mouse primary cortical neurons were treated with each CM for 24 h to assess the cell viability (Fig. 7a). As shown in Fig. 7b, we observed neurotoxicity of LPS-stimulated BV2 CM. However, pre-treatment of NJK14047 in BV2 cells alleviated the neurotoxicity induced by LPS-stimulated BV2 CM (Fig. 7b, left). In contrast, LPS-stimulated C8-D1A CM showed no neurotoxicity (Fig. 7b, right). These cell viability results were confirmed in TUNEL assay; TUNEL-positive neurons were increased in the group treated with LPS-stimulated BV2 CM, but decreased in the NJK14047-treated BV2 CM group (Fig. 7c).

**Fig. 8:**
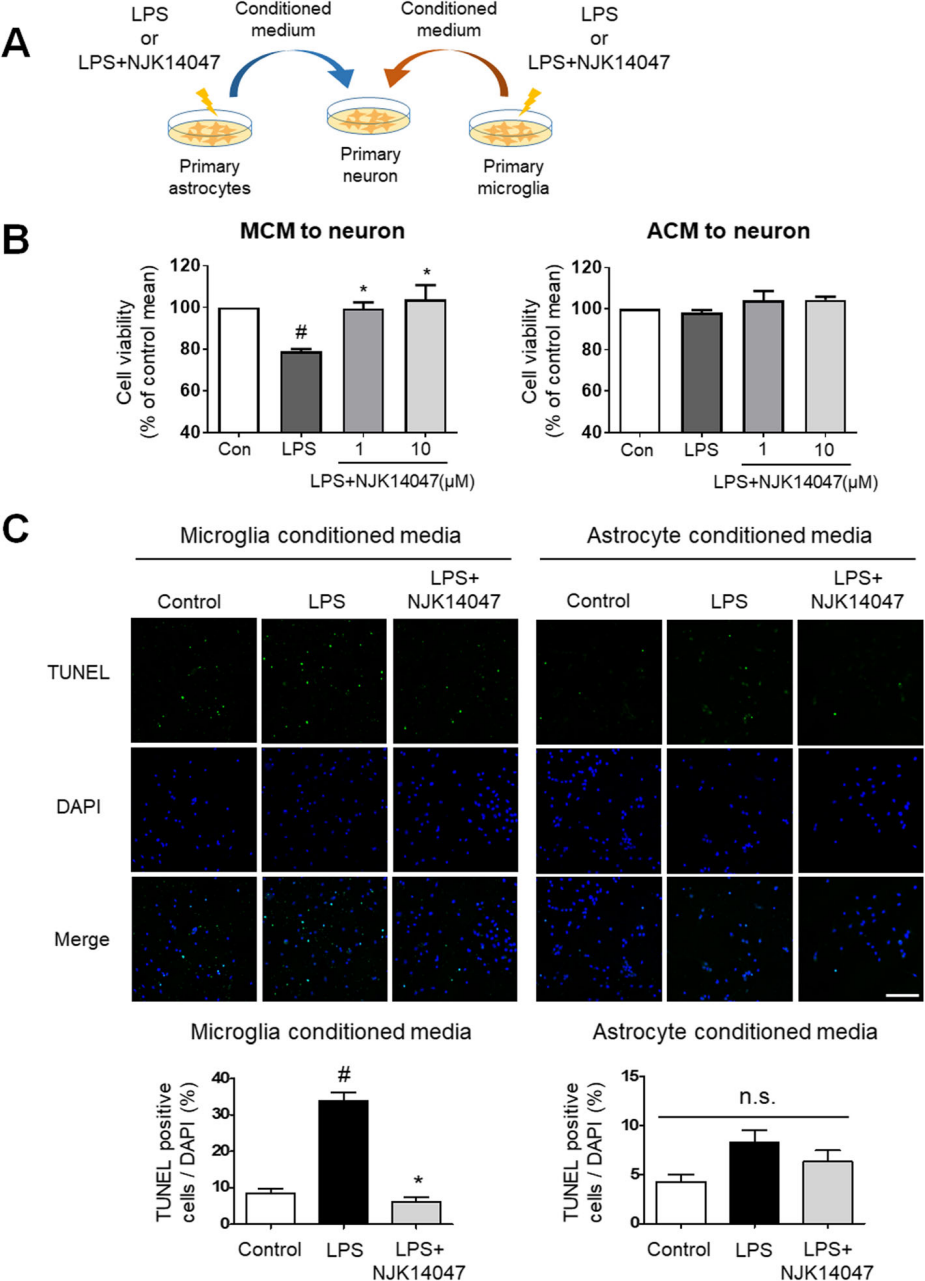
NJK14047 reduces LPS-stimulated microglial neurotoxicity rather than LPS-stimulated astrocyte neurotoxicity. **a** Scheme of the experiment. Mouse primary microglia and astrocytes were seeded on 6-well plate at 5 × 10^5^ cells/well and stimulated with 50 ng/ml LPS alone or after 2 h of pre-treatment with 10 μM NJK14047. After 22 h of LPS stimulation, all media were changed to fresh neurobasal medium where cells were incubated for 24 h. The conditioned medium was collected to treat primary neurons seeded on 24-well cover glass for TUNEL assay. **b** Viability of mouse primary neurons incubated with MCM and ACM for 24 h. O.D. at 570 nm were normalized to the control group and shown as percentage of control mean ± SD (*n* = 3, Kruskal-Wallis test). **c** TUNEL assay for mouse primary neurons stimulated with mouse primary microglia- and astrocyte-conditioned media. Apoptotic cells were quantified using four images in each cover glass. Data are shown as mean ± SEM (scale bar = 100 μm, *n* = 5, one-way ANOVA). ^#^*P* < 0.05 vs. the control group; *P < 0.05 vs. the LPS group

The decreased neurotoxicity of LPS-stimulated BV2 CM with NJK14047 treatment was further confirmed using primary microglia and astrocytes. Primary microglia-conditioned medium (MCM) and astrocyte-conditioned medium (ACM) were prepared as described in the “Methods” section. Primary neurons were incubated with each CM for 24 h and subjected to the MTT and TUNEL assays (Fig. 8a). Similar to our cell line study, LPS-stimulated MCM produced neurotoxicity which was reversed by NJK14047 treatment; however, no changes were observed with ACM (Fig. 8b). Consistent with the MTT assay, more TUNEL-positive cells were detected in the LPS-stimulated MCM group in comparison to the naïve MCM group; NJK14047 treatment resulted in much fewer TUNEL-positive cells than the untreated group. As expected, ACM-treated neurons showed no significant alterations (Fig. 8c). Overall, based on these findings, reactive microglia, rather than astrocytes, provoke the apoptosis of neurons, and NJK14047 can attenuate neurotoxicity of LPS-stimulated MCM.

## Discussion and conclusions

5XFAD mouse is a transgenic AD mouse model which is characterized by rapid accumulation of Aβ in the brain, leading to chronic neuroinflammation. This model exhibits Aβ aggregation and gliosis from 2 months of age, followed by neuronal loss from 6 months of age [24]. 5XFAD mice showed spatial learning memory impairment after 9 months of age [48] and motor function deficits after 12 months of age [49]. In the present study, we showed NJK14047 treatment from 6-month-old 5XFAD mice could significantly ameliorate Aβ-induced neuroinflammation, thereby alleviating neuronal degeneration and cognitive impairment in the 9-month-old 5XFAD mice.

P38 MAPK inhibition is one of the promising targets for AD therapy. Numerous studies have attempted to attenuate Aβ-induced neurotoxicity and memory impairment through inhibition of the p38 MAPK signaling [50, 51]. Recent reports using selective p38α MAPK inhibitors, neflamapimod (VX-745), MW150, and MW181, have shown potential therapeutic effects for AD. Neflamapimod (VX-745) improved water maze performance in aged rats [19] and enhanced episodic memory in early AD patients [21, 22]. In addition, MW150 was reported to show therapeutic effects in APP/PS1 mice at the age of 11 to 12 months [20, 52]. Furthermore, MW181 was suggested to reduce tau-related pathologic features and improve working memory in 20-month-old hTau mice [53]. In this study, we presented the therapeutic effects of a selective p38α/β MAPK inhibitor NJK14047 and investigated its mode of action in 9-month-old 5XFAD mice. P38 MAPKs have been well known to be activated in chronic inflammatory conditions such as AD. Consistently, several studies have shown the upregulation of phospho-p38 levels in the brains of AD mouse models, but not in 5XFAD mice. We demonstrated upregulation of the phospho-p38 levels in the cortex and hippocampus of 9-month-old 5XFAD mice, which could be reversed by NJK14047. Although blood-brain barrier (BBB) is a major obstacle for many drugs targeting the brain, NJK14047 successfully achieved its inhibitory effect on p38 MAPKs in the cortex and hippocampus of 5XFAD mice. In fact, it is plausible that the effect of NJK14047 might be attributed to the damaged BBB of old 5XFAD mice due to chronic inflammatory conditions. The low molecular weight (MW = 445.515) and lipophilic property (cLogP = 3.12) of NJK14047 might also contribute to sufficient penetration into the brain. Further experiments are warranted to determine whether NJK14047 can cross the intact BBB.

Downregulation of the phospho-p38 MAPK level is related to the decreased neuroinflammation, which can reverse reactive gliosis and overexpression of pro-inflammatory cytokines such as TNF-α, IL-1β, and IL-6. According to a recent study using the p38α MAPK inhibitor MW150 in the APP/PS1 mice at the age of 11 to 12 months, IL-1β and TNF-α levels were decreased; microglial cell counts were increased around the plaque [20]. Similarly, we confirmed NJK14047, reduced the pro-inflammatory cytokines, and upregulated alternatively activated microglial markers such as Arg1, YM-1, and Fizz-1 in the cortex and hippocampus of 5XFAD mice. In addition, NJK14047 treatment increased the mRNA expression of microglial phagocytic receptors in the brain and improved the number of plaque-associated microglia, suggesting the recovery of microglial phagocytic activity [54]. In neuroinflammatory conditions, resident microglia or infiltrated macrophages were able to polarize into the pro-inflammatory phenotype (M1) upon exposure to pro-inflammatory cytokines or protein debris. These cells express high levels of pro-inflammatory cytokines and reactive oxygen species, which can be toxic to neurons. In contrast, the alternative activation of microglia (M2) has been previously reported to have positive influences on tissue repair, immunological regulation, and phagocytosis-mediated Aβ clearance [43, 55]. Thus, NJK14047 treatment could alter the ratio of M1 to M2 microglia population, ultimately ameliorating AD pathology. However, this dichotomous classification of in vivo microglia into M1 and M2 may be oversimplified [56]. More specifically, elaborated analysis and classification are required to evaluate the microglial variation with NJK14047 treatment.

The process of generating Aβ peptide from APP could be mediated by the β-secretase and γ-secretase complex; in AD, the produced Aβ is accumulated in the extracellular matrix. Aβ peptides could be degraded by proteolytic enzymes such as IDE, NEP, and MMP-9 or phagocytosed by microglia or astrocytes. Our study demonstrated NJK14047 treatment reduced Aβ accumulation in 9-month-old 5XFAD mice. However, no significant differences in APP processing enzymes or Aβ degradation enzymes were observed with NJK14047 treatment. According to recent studies regarding p38α MAPK in AD mouse models, genetic modulation of neuronal p38α MAPK could ameliorate pathologic features associated with AD [57, 58]. They showed downregulation of p38α MAPK in neurons reduced BACE1 activity, thereby lowering Aβ accumulation in the mice with early-stage AD. Interestingly, Colié et al. reported no significant alterations in the BACE1 protein levels between the experimental groups in 12-month-old 5XFAD mice, possibly indicating variable effects of neuronal p38α inhibition on the BACE1 levels depending on the age of 5XFAD mice [57]. Based on these results, NJK14047 might inhibit the neuronal p38α/β MAPKs and decrease the BACE1 levels in the early phase of treatment; however, these effects might be diminished in 9-month-old mice used in this study. Additionally, our current study showed increased phagocytic receptors of microglia and the number of microglia around Aβ plaque, ameliorating neuroinflammatory conditions. The enhanced microglial phagocytic function might also contribute to the decrease in Aβ accumulation.

Another possible cause of Aβ reduction and restoration of microglial phagocytic function is inhibition of p38β MAPK by NJK14047. According to a previous study, both p38α and p38β MAPKs were highly active in the basal brain condition. However, p38α and p38β showed different localization, indicating their potentially distinct roles or mechanisms. In particular, p38β was detected in glial cells as well as neuronal nuclei in many brain regions [16]. Thus, p38β MAPK inhibition might be important to successfully ameliorate the inflammatory conditions of glial cells, suggesting dual inhibition of both p38α and p38β as an effective therapeutic strategy for AD. Further studies are needed to elaborate this theory.

During the final stage of AD, irreversible neuronal loss occurs in the cortex and hippocampus regions which play a role encoding of new information and consolidation of memory networks. In this regard, protection of neurons against harmful factors such as Aβ plaques or massive pro-inflammatory signals is important for AD therapy. In this study, spatial learning memory was significantly improved in NJK14047-treated 5XFAD mice compared to vehicle-treated 5XFAD mice. Moreover, neuronal degeneration was significantly decreased in the brains of NJK14047-treated mice. Furthermore, the neuroprotective effects of NJK14047 were observed in in vitro studies; NJK14047 treatment reversed the neuronal apoptosis caused by LPS-stimulated BV2 CM. Interestingly, LPS-stimulated C8-D1A CM showed no toxicity to the tested neurons. This neuroprotective effect of NJK14047 was further investigated using primary microglia and astrocytes. Consistent with cell line studies, apoptosis occurred in the neurons cultured in the LPS-stimulated MCM; however, this neurotoxicity was alleviated by pre-treatment with NJK14047. Similar to C8-D1A, LPS-stimulated ACM showed no apparent toxicity to neurons. Although recent studies have reported the toxic effects of reactive astrocytes with A1 phenotype on neurons, polarization into A1 astrocytes requires activated MCM including IL-1α, TNF, and C1q [59]. Collectively, LPS-stimulated astrocytes without pro-inflammatory microglial activation are suggested to be less toxic to neurons, and inhibition of microglial activation by NJK14047 may contribute to neuroprotection.

In summary, we have shown pharmacological inhibition of p38α/β MAPKs in the brain of 5XFAD mice contributes to reduction of Aβ accumulation as well as amelioration of neuroinflammatory conditions and microglial phagocytic functions; as a result, neuronal death was decreased, and learning memory was improved. As NJK14047 generally acts on neurons and glia by inhibiting both p38α and p38β MAPKs, further studies are warranted to evaluate which p38 MAPK accounts for the therapeutic effects of NJK14047 in neurons and glia. Taken together, a selective p38α/β MAPK inhibitor NJK14047 successfully showed therapeutic effects in 5XFAD mice. Our data support the potential of p38 MAPK inhibition as a treatment strategy for AD, suggesting NJK14047 might be one of the promising candidates for AD therapeutics targeting p38 MAPKs.

## Data Availability

The datasets used and/or analyzed during the current study are available from the corresponding author on reasonable request.

## Abbreviations

Aβ: Amyloid beta
ACM: Astrocyte-conditioned medium
AD: Alzheimer’s disease
Arg1: Arginase 1
BACE1: Beta-site APP cleaving enzyme 1
BBB: Blood-brain barrier
Chi3l3/YM-1: Chil3 chitinase-like 3
CM: Conditioned medium
GFAP: Glial fibrillary acidic protein
hAPP: Human amyloid precursor protein
Iba-1: Ionized calcium-binding adapter molecule 1
IDE: Insulin degrading enzyme
MACRO: Macrophage receptor with collagenous structure
MCM: Microglia-conditioned medium
Msr1: Macrophage scavenger receptor 1
NEP: Neprilysin
PS1: Presenilin 1
Retnla/Fizz-1: Resistin-like alpha
Scarb1: Scavenger receptor class B, member 1
Scarb3: Scavenger receptor class B, member 3
